# Targeting microRNA-122 to Treat Hepatitis C Virus Infection

**DOI:** 10.3390/v2071382

**Published:** 2010-07-05

**Authors:** Catherine L. Jopling

**Affiliations:** School of Pharmacy, Centre for Biomolecular Sciences, University of Nottingham, University Park, Nottingham NG7 2RD, UK; E-Mail: catherine.jopling@nottingham.ac.uk

**Keywords:** microRNA-122, hepatitis C virus

## Abstract

An important host factor for hepatitis C virus (HCV) is microRNA-122 (miR-122). miR-122 is a liver-specific member of a family of small, non-coding RNA molecules known as microRNAs that play major roles in the regulation of gene expression by direct interaction with RNA targets. miR-122 binds directly to two sites in the 5′ untranslated region (UTR) of HCV RNA and positively regulates the viral life cycle. The mechanism by which this regulation occurs is still not fully understood. There has been a great deal of interest in potential therapeutics based on small RNAs, and targeting miR-122 to combat HCV is one of the furthest advanced. Chemical inhibitors of miR-122 can be introduced into mammals intravenously and result in potent and specific knockdown of the microRNA, with no detectable adverse effects on liver physiology. This strategy was recently applied to chimpanzees chronically infected with HCV and resulted in a sustained reduction in viral load in the animals. Inhibition of miR-122 therefore presents a very attractive novel approach to treating HCV, a virus for which improved therapeutics are urgently needed.

## 1. microRNAs: versatile regulators of gene expression

In the last 10 years there have been enormous advances in our understanding of the role of small non-coding RNA molecules in the regulation of eukaryotic gene expression. microRNAs (miRNAs) are a particularly important class of small RNA. They have been implicated in various human diseases, including a number of viral infections, suggesting that future therapeutics may be able to exploit aspects of this biology. This review concerns the role for a specific miRNA, miR-122, in the HCV life cycle, and its potential as an anti-HCV target.

miRNAs are 21–23 nucleotide (nt) non-coding RNA molecules that are expressed by a broad range of eukaryotic species [1]. They are encoded in the genome and transcribed, usually by RNA polymerase II, as part of long primary miRNAs (pri-miRNAs). These may be unique transcripts, introns of coding mRNAs, or polycistronic transcripts containing a cluster of miRNAs [2]. Pri-miRNAs undergo nuclear processing by the Microprocessor complex to generate a precursor miRNA (pre-miRNA). Pre-miRNAs are subject to nuclear export, and cytoplasmic processing by the enzyme Dicer, to yield the mature, single-stranded miRNA [2]. In the most recent release of miRBase (version 15), 940 different miRNAs have been identified in humans [3]. In higher organisms, miRNA expression is specialized according to both tissue type and developmental stage [4], and de-regulation of expression of certain miRNAs is associated with cancer [5].

There is considerable overlap between the miRNA pathway and the process of RNA interference (RNAi). RNAi is induced by long, double-stranded RNA, which is processed by Dicer to produce 21–23 nt RNA duplexes known as short interfering RNAs (siRNAs) [6]. Both miRNAs and siRNAs associate with a complex of proteins that is necessary for their function. This is known as the RNAi-induced silencing complex (RISC), and the siRNA and miRNA-directed complexes are designated siRISC and miRISC, respectively. miRNAs that encounter exactly complementary targets act similarly to siRNAs and cleave the target RNA. This is the major mechanism of miRNA activity in plants, but very unusual in animals; however, this interchangeability of function indicates that the miRISC and siRISC are likely to share many common features [6]. The exact identity of the two RISCs is not certain, and it is possible that specialized versions may exist. A member of the argonaute protein family is an essential component; there are four such proteins in mammals (Ago1-4), of which only Ago2 can mediate RNA cleavage. Any of the four Ago proteins appear to be able to mediate miRNA activity in mammals, which also requires one of the three GW182 proteins (TNRC6A-C) [1].

Most animal miRNAs act by binding to imperfectly complementary target sites. Several algorithms have been used to predict miRNA target sites with varying degrees of success. Analysis of target sites has revealed some indicators of functional sites, most notably direct complementarity to the miRNA ‘seed’, comprising nt 2–7 or 2–8 from the 5′ end [7]. Animal miRNAs usually bind to sites in the 3′ untranslated region (UTR) of mRNAs, resulting in reduced production of the corresponding protein [7]. There is still considerable debate about the underlying mechanism. Different groups have observed translation repression either at initiation or post-initiation, and RNA degradation is also apparent [1]. Current evidence would suggest that the translation block occurs at the level of initiation and that RNA degradation occurs as a consequence of this [8], but it is possible that different miRNA-target pairs or cellular conditions may result in different consequences.

There are many examples of interaction between the miRNA pathway and viral infection. A number of DNA viruses, in particular the herpesviruses, encode their own miRNAs [9]. These viral miRNAs repress viral or cellular targets and may be important in establishing patterns of latent or lytic infection. Viral infection can also regulate the expression of cellular miRNAs [10]. There is evidence that cellular miRNAs can bind to target sites in certain viruses, notably human immunodeficiency virus-1 (HIV-1), and repress production of the virus, but it is not certain whether this is really a physiologically relevant antiviral mechanism [9]. There is currently only one known example of a cellular miRNA that has a direct, positive effect on a viral life cycle; this is the interaction between miR-122 and HCV.

## 2. miR-122 and HCV

miR-122 is conserved, both in sequence and in its liver-specific expression pattern, from humans to zebrafish [4,11]. It accounts for around 70% of the total miRNA content of liver [4]. HCV is a positive sense RNA virus that infects the liver, frequently establishing persistent infections that can eventually result in liver cirrhosis and carcinoma [12]. The HCV RNA genome is 9.6 kilobases (kb) in length and consists of a single coding region flanked by structured 5′ and 3′ UTRs that are important for replication [13]. Translation of the viral polyprotein is driven by an internal ribosome entry site (IRES) in the 5′ UTR of the viral RNA. This IRES is unusual in its ability to recruit the 40S ribosomal subunit directly and initiate protein synthesis using a minimal subset of eukaryotic translation initiation factors [14]. The polyprotein is cleaved by viral and cellular proteases to produce mature structural and nonstructural proteins. Replication of the viral RNA then proceeds via a negative strand intermediate in membrane-bound replication complexes [13].

**Figure 1:**
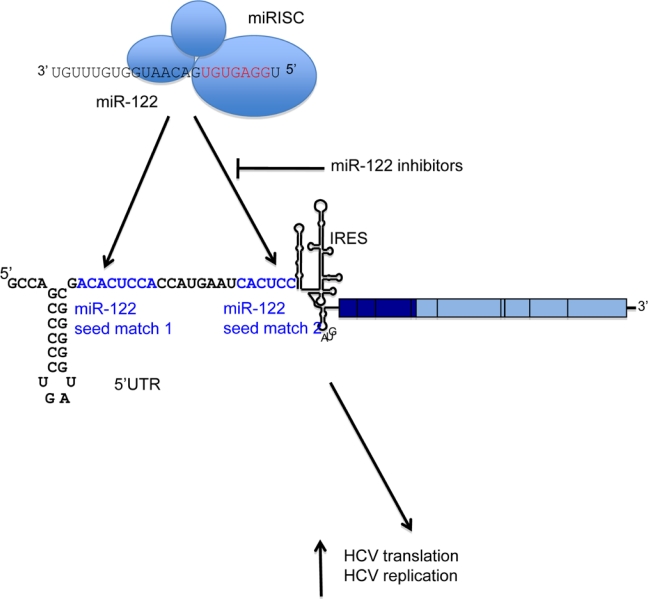
A schematic of the HCV RNA genome is shown, with the miR-122 binding region expanded. miR-122, in collaboration with the RISC, binds directly to two adjacent seed matches (highlighted in blue) in the HCV 5′ UTR. This results in activation of both HCV translation and a further, unidentified stage of the replication cycle. Inhibitors can be directed against miR-122 to block binding to its target sites and reduce HCV replication.

The role for miR-122 in HCV infection was first demonstrated by sequestration of endogenous miR-122, which led to a substantial reduction in HCV RNA in human liver cells that contain HCV replicons [15]. This is due to a direct interaction between miR-122 and two adjacent binding sites, which both have seed match complementarity to the miRNA [16]. Interestingly, these binding sites are in the 5′ UTR of the viral RNA (Figure 1). They are located in a single-stranded region of RNA immediately upstream of the HCV IRES, and are conserved across HCV genotypes. Mutagenesis of the miR-122 binding sites indicates that both sites are necessary for HCV replication to occur efficiently [16]. In an infectious HCV system, site 1 is absolutely required for infection, whereas the requirement for site 2 can be overcome by overexpression of miR-122 [17].

It is not yet clear how miR-122 regulates HCV, although a positive role for RISC components in an infectious virus cell culture system suggests that this is a RISC-dependent process [18]. There has been some debate about whether or not viral translation is activated by the miRNA [15,19]. A recent study by Jangra *et al.* demonstrated that miR-122 does mediate a positive effect on HCV translation, but that this is not sufficient to explain its effects on replication of the virus [17]. This implies that a second mechanism of regulation is in play. The identity of this mechanism remains elusive, as miR-122 had little effect on new HCV RNA synthesis when this was measured directly by thiouridine labeling [20]. It is possible that an effect on viral RNA turnover may be involved, or that a role for miR-122 at a specific stage of the viral life cycle has been missed by previous experiments. A better understanding of the mechanistic details of the interaction between miR-122 and HCV will be important when considering miR-122 as a target for HCV therapy.

HCV infection is currently treated with a combination of pegylated interferon-α and ribavirin. This is poorly tolerated and ineffective in a high proportion of patients, and novel therapies are urgently needed [12]. The role for miR-122 as an important, possibly essential, host factor for HCV makes it an attractive target for antiviral therapy. The rapid evolution of RNA viruses means that the development of resistance is frequently associated with the direct targeting of viruses. Using a host factor as a target has the potential to avoid this problem. The recent development of effective miRNA inhibitors that can be administered *in vivo* suggested that direct inhibition of miR-122 would be a good strategy for a new HCV therapy.

## 3. miRNA inhibition as a therapeutic tool

The study of miRNAs has been greatly facilitated by the use of antisense oligonucleotides as inhibitors. The first application of this approach used an RNA oligomer complementary to the miRNA *let-7*, with 2′-O-methyl (2′-OMe) modifications at each position [21]. 2′-OMe RNA was chosen because it is resistant to cellular ribonucleases, and because it is bound by the complementary miRNA in association with the RISC. This is a crucial feature for the use of such molecules as inhibitors; it provides much greater efficacy than would be possible with simple hybridization to a complementary target, it allows the use of low (nanomolar) quantities of the inhibitor, greatly reducing the possibility of off-target effects, and the sequestration of the miRNA in its functional complex prevents its interaction with target RNA in a stable fashion. This oligomer effectively prevented *let-7* action *in vitro*, in cultured HeLa cells following transfection using a liposomal reagent, and in *C. elegans* after injection. The molecule was non-toxic in *C. elegans* [21].

The characteristics of these 2′-OMe inhibitors – notably tight binding at low concentration, specificity of action, and lack of associated toxicity – suggest that inhibition of miRNAs by similar methods might be a promising avenue for drug development. The success of this method in *C. elegans* is also important as it suggests that inhibition is possible in whole animals. This strategy, therefore, has much potential for therapeutics, as the expression of specific miRNAs is intimately connected with development and cell physiology, and therefore with disease. The greatest advances in this area have been directed at reducing HCV viral load by targeting miR-122, the focus of this review. However, miRNA-based medicines have much broader potential, particularly in cancer treatment, as there is a strong association between expression of certain miRNAs and development of certain tumors [5].

Since the initial studies with 2′-OMe oligomers, different chemistries have been tested to try to optimize binding affinity, stability and uptake of similar miRNA inhibitors. Several different 2′ sugar modifications that should increase binding affinity were tested in an assay for miR-21 inhibition. Locked nucleic acid (LNA), 2′-O-methoxyethyl (2′-MOE), or 2’-fluoro (2′-F) modifications to the sugar all improved the inhibition properties of the oligomer when compared to a 2′-OMe modification [22]. The effect on the miRNA varies according to the inhibitor used. LNA-modified oligomers form stable complexes with the target miRNA, whereas some other modifications result in degradation of the target, and some do not [23]. Functional readouts are therefore necessary to establish the efficiency of inhibition. Different sugar modifications have been combined to create inhibitors with improved properties. A 2′-F/MOE-modified oligomer was the most effective of several molecules tested for inhibition of miR-122 in cell culture [23]. LNA modification imparts increased binding affinity and specificity when incorporated at some positions in a DNA oligonucleotide. 16 nt oligomers with a mixture of DNA and LNA bases have been successfully used to target miR-122 *in vivo* [24–26].

Delivery of miRNA inhibitors to tissues presents technical challenges. Carriers such as liposomes are widely used in cell culture to protect the oligonucleotide from degradation and allow delivery across the hydrophobic cell membrane [27]. Similar delivery methods could be used *in vivo*, but successful delivery to the liver has been possible without packaging. A cholesterol-conjugated antisense oligonucleotide, known as an ‘antagomir’, directed against miR-122 was administered by intravenous injection and was delivered to the liver [28]. Peptide nucleic acid (PNA) oligomers coupled to a cell-penetrating peptide or four lysine residues could enter cells without transfection reagent and inhibit miR-122 in cell culture [29]. A PNA-based, lysine-conjugated oligomer was also used to inhibit the oncogenic miR-155 in B cells *in vivo* [30].

Successful systemic delivery of antisense oligonucleotides has also been accomplished without conjugation by incorporating a phosphorothioate backbone. Phosphorothioate modification improves the stability of the oligomer, delays plasma clearance, and allows entry into tissues [31]. miR-122 has been targeted by this method using a 2′-MOE inhibitor administered by intraperitoneal injection. A phosphorothioate backbone was also used in the LNA-based miR-122 inhibitors, and allowed very effective miR-122 inhibition following intravenous injection [24–26].

Systemic delivery by intravenous injection is desirable, and the success with these miR-122 inhibitors indicates that it is possible to target liver miRNAs by this method. The liver specificity of miR-122 expression means that side effects of drug delivery to other tissues are not likely to be a problem, unless the drug has non-specific toxic effects. Different miRNAs in other tissues are likely to prove more difficult to target and may require localized delivery mechanisms, or different strategies [27]. One possibility is to use miRNA ‘sponges’, DNA plasmids encoding an RNA that contains multiple copies of a target for a particular miRNA. These RNAs bind the endogenous miRNA and reduce its availability for endogenous targets [32]. As sponges are DNA-based, they have the potential for introduction into cells by viral delivery, which could be very useful when targeting certain tissues. However, this method is not as effective or simple as direct inhibition of the miRNA.

## 4. Successful inhibition of miR-122 in animals

The sequence of miR-122 is completely conserved in mammals, which has allowed optimization of targeting strategies in animal models. Inhibition of miR-122 in mice was first accomplished using an ‘antagomir’ [28] and a 2′-MOE-modified oligomer with a phosphorothioate backbone [33]. Both studies yielded similar results.

An obvious concern when targeting a miRNA is that this will result in changes in expression of its endogenous targets, with potentially damaging consequences. Encouragingly, the animals in these studies did not show any evidence of liver toxicity, but did experience a substantial reduction in plasma cholesterol [28]. Microarray analysis was used to identify mRNAs that change in expression when miR-122 is inhibited. A high proportion of mRNAs that increased on miR-122 inhibition have seed matches for miR-122 in the 3′ UTR, and are therefore likely to be direct endogenous targets for the miRNA. Several mRNAs encoding proteins that are involved in cholesterol synthesis were downregulated on miR-122 inhibition, suggesting that this pathway is regulated indirectly. miR-122 inhibitors therefore have potential therapeutic value in both HCV and cholesterol disorders.

miR-122 inhibition in mice was also achieved using an inhibitor containing an optimized combination of DNA and LNA-modified bases complementary to nt 1–16 of miR-122, with a phosphorothioate backbone [24]. The LNA inhibitor was delivered to murine liver following intravenous injection, and effective sequestration of miR-122 was observed by formation of a heteroduplex with the inhibitor, increases in the mRNA level of endogenous miR-122 targets, and a reduction in plasma cholesterol. The doses required were much lower than in the antagomir experiments, so an LNA-based strategy seems the most promising approach to take for future therapeutics.

LNA-based oligomers were then tested in a non-human primate, the African green monkey. The results obtained were very similar to those in mice, with formation of miR-122-inhibitor complexes, increases in the level of endogenous targets, a reduction in plasma cholesterol, and no detectable liver toxicity [25]. The animals were treated with three intravenous injections and observed over the course of several weeks. Plasma cholesterol reduction was sustained for five to seven weeks, and then returned to pre-treatment levels as LNA-antimiR-122 was cleared from the liver. The results of this study were encouraging for therapeutic development, firstly because the inhibitor shows similar effects and similar lack of toxicity in primates as in mice, implying that it could also be effective in humans, and secondly because the effects of the inhibitor are sustained and reversible, which are desirable properties for a drug.

## 5. miR-122 inhibition reduces HCV viral load in chimpanzees

LNA-antimiR-122, now known as SPC3649, has recently been used to inhibit miR-122 in chimpanzees chronically infected with HCV [26]. The animals were treated at weekly intervals over a 12-week period. Excitingly, three out of the four animals treated showed a sustained reduction in viral load. The fourth animal had fluctuations in viral load throughout the study, making it difficult to assess. This was one of two animals that received a low dose (1 mg/kg). This was considerably less effective than the high dose (5 mg/kg); even the animal that responded to the low dose only had a reduction in liver HCV RNA of 1.3 orders of magnitude, compared to 2.3 orders of magnitude in HCV RNA in the liver, and 2.6 in the serum, of the high dose animals [26]. The reasons for this are not clear, as both dosing regimes resulted in sequestration of all detectable miR-122 into a heteroduplex with the inhibitor. It is possible that undetectable levels of miR-122 remaining after the low dose therapy were sufficient to support HCV replication; we know little about the stoichiometry of the miR-122-HCV interaction in infected liver. There was no significant change in expression of endogenous miR-122 target genes in the non-responder, supporting the theory that miR-122 was not effectively targeted in this animal [26]. Heteroduplex formation between miR-122 and the inhibitor is therefore not a very reliable method of assessing the efficiency of inhibition.

The reduction in HCV RNA was sustained over the course of treatment, and was gradually lost once the inhibitor was withdrawn. The miR-122 binding region of HCV RNA was examined by deep sequencing in samples taken from the high dose animals before, during and after therapy. No adaptive mutations were detected in this region, which is very encouraging [26]. This suggests that miR-122 cannot be replaced by a different miRNA, which is supported by the previous observation that mutation of miR-122 seed match 1 to a miR-21 seed match does not allow miR-21 to regulate HCV [16]. It is possible that mutations might arise elsewhere in the HCV genome, or over a longer time-course of treatment, which would allow viremia to rebound. However, the contrast with the rapid evolution of mutations to most drugs that directly target HCV is striking.

The chimpanzees showed a similar increase in levels of miR-122 target mRNAs and reduction in total plasma cholesterol to the other animals that have been treated. One interesting difference was that the cholesterol reduction in chimpanzees was predominantly in low-density lipoprotein (LDL) and apolipoprotein Apo-B, in contrast to African green monkeys where high-density lipoprotein (HDL) and its apolipoprotein Apo-A1 were mainly affected [25,26]. It is probable that the results in chimpanzees will be more representative of human patients, so these results are encouraging when considering miR-122 targeting for cholesterol reduction, as high LDL levels are strongly associated with cardiovascular disease [34]. Numerous indicators of liver toxicity were examined, and no problems were detected. The only change observed was an improvement in overall liver histology in the high dose animals that accompanied the reduction in viral load [26].

The HCV-infected chimpanzee model has several significant differences to human infection. Chimpanzees typically experience milder symptoms than human patients, and do not progress to cirrhosis [35]. Chronically infected chimpanzees have a permanently induced interferon system that does not respond to stimuli. In this, they are similar to human interferon non-responders, who have elevated levels of interferon and do not respond to interferon therapy. One very interesting result of this study was a reduction in expression of interferon-regulated genes (IRGs) accompanying the reduction in viral load in the animals that received the high dose therapy, or responded to the low dose [26]. This is encouraging as it suggests that miR-122 targeting might be effective in restoring a functional interferon system to non-responding patients, even if the miR-122 inhibitor itself does not fully eliminate infection.

## 6. Outlook for miRNA-based therapies against HCV

The effects of miR-122 inhibition on HCV infection in chimpanzees suggest that this method could provide a very exciting novel approach to HCV therapy.

Lanford *et al*’s results have greatly increased our knowledge from previous cell culture studies by showing that there is a correlation between miR-122 activity and HCV RNA levels in infected chimpanzees [26]. It is reasonable to assume that SPC3649 represses HCV infection in animals by directly preventing the interaction between miR-122 and HCV RNA, as all evidence from cell culture indicates that this is the only mechanism by which miR-122 regulates HCV. Replication of HCV is lost completely when the miR-122 binding sites are mutated, but can be fully restored by supplementation with a mutant miR-122 that binds the mutant site [15–17]. This implies that the role for miR-122 in HCV infection is entirely dependent on its direct interaction with its binding sites. In support of this, regulation of HCV by miR-122 was shown to be independent of the effects of miR-122 on isoprenoid biosynthesis and the requirement for products of this pathway in HCV replication [20]. The lack of evolution of adaptive mutations and sustained response to miR-122 sequestration in infected chimpanzees suggests that the miR-122-HCV interaction is essential for the virus and cannot easily be overcome [26].

The exciting results in chimpanzees provide an important starting point for taking this inhibitor into human trials, but it is difficult to predict how effective it may be. The chimpanzee research was on too small a scale to draw any statistical conclusions, and it is important to bear in mind the differences in infection patterns in chimpanzees and humans. Analysis of miR-122 levels in HCV-infected humans suggests that the relationship between the miRNA and the virus may be rather more complex than in cultured cells. Levels of miR-122 were quantified in liver biopsies taken from patients, and showed no positive correlation with viral load [36]. Moreover, patients who did not respond to pegylated interferon-α therapy had lower miR-122 expression at the start of treatment than patients who showed a sustained virological response. Interferon treatment did not affect miR-122 expression [36].

The minor reduction in HCV RNA in chimpanzees receiving a low dose of miR-122 inhibitor may have bearing on these observations, as this suggests that a very low level of miR-122 may be sufficient to support HCV replication *in vivo* [26]. miR-122 expression in the liver is four-fold higher than in cultured Huh-7 cells [37], which support highly efficient HCV replication, so the reduced levels of miR-122 in the interferon non-responders could be adequate for viral replication to occur. It is even possible that lower endogenous miR-122 in non-responding patients would make them more susceptible to miR-122 inhibitors as HCV therapy. The changes in interferon levels in chimpanzees subjected to SPC3649 treatment are also intriguing in this context, as this raises the possibility that miR-122 sequestration could restore an interferon response by reducing HCV load [26].

One very encouraging feature of miR-122 inhibitors as anti-HCV therapeutics is the lack of liver toxicity. A major problem with the current pegylated interferon-α/ribavirin therapy is the associated side effects, which reduce adherence to the regime [12]. In contrast to this, the reduction in plasma cholesterol in response to miR-122 sequestration would actually benefit patients. However, miR-122 is a highly conserved, highly expressed miRNA that regulates numerous cellular targets. The consequences of interfering with its activity in the long term could easily be problematic, particularly considering the association between altered miRNA regulation and cancer [5]. It will be necessary to follow up the treated chimpanzees to detect any long term effects that do arise. It would seem wisest to apply the miR-122 inhibitor over fairly short time periods, in collaboration with other drugs that target other aspects of the viral life cycle, with the aim of rapidly clearing HCV infection. Alternatively, it is possible that miR-122 antagonism could be used to restore interferon non-responders to a state in which pegylated interferon-α/ribavirin can be used effectively [26].

If miR-122 inhibitors do prove effective as anti-HCV drugs in humans, it is almost certain that this will be as part of a combined therapy. The reduction in viremia in the treated chimpanzees was substantial but came some way short of clearing the virus, and the rebound that occurred when the inhibitor was withdrawn suggests that inhibition of miR-122 alone is never likely to be sufficient. This is an exciting time for HCV research, with particular progress in the use of HCV protease inhibitors. Two such inhibitors, telaprevir and boceprevir, are now in advanced clinical trials and have shown promising viral clearance rates [38]. However, resistance to the inhibitors has been detected, so there is still a need for new drugs for which this is less of a problem. Both trials combine the protease inhibitors with pegylated interferon-α/ribavirin, so toxicity remains a problem and makes the treatment unsuitable for many patients [38]. The development of different combination therapies based solely on small molecules is desirable. Anti-miR-122 will be an important contributor to these combined therapies because evolution to avoid its effects does not seem to occur [26].

The LNA and phosphorothioate-modified SPC3649 appears to possess all the necessary characteristics of a highly effective, non-toxic, miR-122 inhibitor in animals [26]. This inhibitor, or a very similar molecule, is the most likely candidate to be used in any anti-HCV therapy based on targeting miR-122. Santaris Pharma is intending to take this molecule, now known as miravirsen, into phase II clinical trials in late 2010, following completion of a phase I dose escalation trial in healthy adult males. A consortium between Regulus and Glaxo Smith Kline is also planning clinical trials to start in 2011 using an antisense oligonucleotide approach to target miR-122, but has not yet identified a candidate molecule for clinical development. Other methods of miRNA inhibition are also possible, and developments in these strategies in the future may provide useful approaches to target this miRNA, perhaps over different timescales or as part of different drug combinations.

In conclusion, there are several features of miR-122 inhibition that make it highly attractive as a means of targeting HCV infection. The conservation of the miR-122 binding sites across all HCV genotypes is important; theoretically, inhibitors would be expected to be equally efficient in treatment of any infection, although in reality it is likely that other features of individual infections will modulate the response to anti-miR-122. HCV shows a strong requirement for the miR-122 interaction in cell culture in both infectious virus and replicon systems, based on different genotypes [15,17,18]. The recent research in chimpanzees demonstrates that miR-122 is also important in HCV infection in animals [26]. Targeting a conserved host factor avoids many of the problems of viral evasion of drug therapy. miR-122 can be effectively inhibited by small molecules that are administered intravenously, and inhibition appears to be non-toxic. The most closely examined inhibitor, SPC3649, shows good pharmacokinetics with sustained, reversible inhibition of the miRNA [26]. The results of human trials of miR-122 inhibitors will be very interesting.

## References

[1] Filipowicz W, Bhattacharyya SN, Sonenberg N (2008). Mechanisms of post-transcriptional regulation by microRNAs: are the answers in sight?. Nat Rev Genet.

[2] Kim VN, Nam JW (2006). Genomics of microRNA. Trends Genet.

[3] Griffiths-Jones S, Saini HK, van Dongen S, Enright AJ (2008). miRBase: tools for microRNA genomics. Nucleic Acids Res.

[4] Lagos-Quintana M, Rauhut R, Yalcin A, Meyer J, Lendeckel W, Tuschl T (2002). Identification of tissue-specific microRNAs from mouse. Curr Biol.

[5] Croce CM (2009). Causes and consequences of microRNA dysregulation in cancer. Nat Rev Genet.

[6] Carthew RW, Sontheimer EJ (2009). Origins and Mechanisms of miRNAs and siRNAs. Cell.

[7] Bartel DP (2009). MicroRNAs: target recognition and regulatory functions. Cell.

[8] Fabian MR, Mathonnet G, Sundermeier T, Mathys H, Zipprich JT, Svitkin YV, Rivas F, Jinek M, Wohlschlegel J, Doudna JA (2009). Mammalian miRNA RISC recruits CAF1 and PABP to affect PABP-dependent deadenylation. Mol Cell.

[9] Umbach JL, Cullen BR (2009). The role of RNAi and microRNAs in animal virus replication and antiviral immunity. Genes Dev.

[10] Skalsky RL, Cullen BR (2010). Viruses, microRNAs, and Host Interactions. Annu Rev Microbiol.

[11] Kloosterman WP, Wienholds E, de Bruijn E, Kauppinen S, Plasterk RH (2006). *In situ* detection of miRNAs in animal embryos using LNA-modified oligonucleotide probes. Nat Methods.

[12] Thomson BJ (2009). Hepatitis C virus: the growing challenge. Br Med Bull.

[13] Lindenbach BD, Rice CM (2005). Unravelling hepatitis C virus replication from genome to function. Nature.

[14] Pestova TV, Shatsky IN, Fletcher SP, Jackson RJ, Hellen CU (1998). A prokaryotic-like mode of cytoplasmic eukaryotic ribosome binding to the initiation codon during internal translation initiation of hepatitis C and classical swine fever virus RNAs. Genes Dev.

[15] Jopling CL, Yi M, Lancaster AM, Lemon SM, Sarnow P (2005). Modulation of hepatitis C virus RNA abundance by a liver-specific MicroRNA. Science.

[16] Jopling CL, Schütz S, Sarnow P (2008). Position-dependent function for a tandem microRNA miR-122-binding site located in the hepatitis C virus RNA genome. Cell Host Microbe.

[17] Jangra RK, Yi M, Lemon SM (2010). miR-122 Regulation of Hepatitis C Virus Translation and Infectious Virus Production. J Virol.

[18] Randall G, Panis M, Cooper JD, Tellinghuisen TL, Sukhodolets KE, Pfeffer S, Landthaler M, Landgraf P, Kan S, Lindenbach BD (2007). Cellular cofactors affecting hepatitis C virus infection and replication. Proc Natl Acad Sci U S A.

[19] Henke JI, Goergen D, Zheng J, Song Y, Schuttler CG, Fehr C, Junemann C, Niepmann M (2008). microRNA-122 stimulates translation of hepatitis C virus RNA. EMBO J.

[20] Norman KL, Sarnow P (2010). Modulation of hepatitis C virus RNA abundance and the isoprenoid biosynthesis pathway by microRNA miR-122 involves distinct mechanisms. J Virol.

[21] Hutvágner G, Simard MJ, Mello CC, Zamore PD (2004). Sequence-specific inhibition of small RNA function. PLoS Biol.

[22] Davis S, Lollo B, Freier S, Esau C (2006). Improved targeting of miRNA with antisense oligonucleotides. Nucleic Acids Res.

[23] Davis S, Propp S, Freier SM, Jones LE, Serra MJ, Kinberger G, Bhat B, Swayze EE, Bennett CF, Esau C (2009). Potent inhibition of microRNA *in vivo* without degradation. Nucleic Acids Res.

[24] Elmen J, Lindow M, Silahtaroglu A, Bak M, Christensen M, Lind-Thomsen A, Hedtjarn M, Hansen JB, Hansen HF, Straarup EM (2008). Antagonism of microRNA-122 in mice by systemically administered LNA-antimiR leads to up-regulation of a large set of predicted target mRNAs in the liver. Nucleic Acids Res.

[25] Elmen J, Lindow M, Schütz S, Lawrence M, Petri A, Obad S, Lindholm M, Hedtjarn M, Hansen HF, Berger U (2008). LNA-mediated microRNA silencing in non-human primates. Nature.

[26] Lanford RE, Hildebrandt-Eriksen ES, Petri A, Persson R, Lindow M, Munk ME, Kauppinen S, Ørum H (2010). Therapeutic silencing of microRNA-122 in primates with chronic hepatitis C virus infection. Science.

[27] Seto AG (2010). The road toward microRNA therapeutics. Int J Biochem Cell Biol.

[28] Krützfeldt J, Rajewsky N, Braich R, Rajeev KG, Tuschl T, Manoharan M, Stoffel M (2005). Silencing of microRNAs *in vivo* with ‘antagomirs’. Nature.

[29] Fabani MM, Gait MJ (2008). miR-122 targeting with LNA/2′-O-methyl oligonucleotide mixmers, peptide nucleic acids (PNA), and PNA-peptide conjugates. RNA.

[30] Fabani MM, Abreu-Goodger C, Williams D, Lyons PA, Torres AG, Smith KG, Enright AJ, Gait MJ, Vigorito E (2010). Efficient inhibition of miR-155 function *in vivo* by peptide nucleic acids. Nucleic Acids Res.

[31] Esau CC (2008). Inhibition of microRNA with antisense oligonucleotides. Methods.

[32] Ebert MS, Neilson JR, Sharp PA (2007). MicroRNA sponges: competitive inhibitors of small RNAs in mammalian cells. Nat Methods.

[33] Esau C, Davis S, Murray SF, Yu XX, Pandey SK, Pear M, Watts L, Booten SL, Graham M, McKay R (2006). miR-122 regulation of lipid metabolism revealed by *in vivo* antisense targeting. Cell Metab.

[34] Preiss D, Sattar N (2009). Lipids, lipid modifying agents and cardiovascular risk: a review of the evidence. Clin Endocrinol (Oxf).

[35] Boonstra A, van der Laan LJ, Vanwolleghem T, Janssen HL (2009). Experimental models for hepatitis C viral infection. Hepatology.

[36] Sarasin-Filipowicz M, Krol J, Markiewicz I, Heim MH, Filipowicz W (2009). Decreased levels of microRNA miR-122 in individuals with hepatitis C responding poorly to interferon therapy. Nat Med.

[37] Chang J, Nicolas E, Marks D, Sander C, Lerro A, Buendia MA, Xu C, Mason WS, Moloshok T, Bort R, Zaret KS, Taylor JM (2004). miR-122, a mammalian liver-specific microRNA, is processed from *hcr* mRNA and may downregulate the high affinity cationic amino acid transporter CAT-1. RNA Biology.

[38] Nelson DR (2009). Hepatitis C drug development at a crossroads. Hepatology.

